# An Essay on the Treatment of the Nerves and the Lining Membrane of the Teeth, When Denuded

**Published:** 1824-03

**Authors:** Leonard Koecker

**Affiliations:** Surgeon-Dentist, Doctor of Medicine and Surgery of the University of Erlangen.


Art. V.—*An Essay on the Treatment of the Nerves and the lining Membrane of the Teeth, when denuded.* By Leonard Koecker, Surgeon-Dentist, Doctor of Medicine and Surgery of the University of Erlangen.*

Among the various difficulties which the dentist has to encounter in the treatment of many complicated diseases of the mouth, are those cases where the teeth are found in so carious a state, that, in cutting away the dead and inflamed parts, with a view of preserving them by filling up the cavity with some metal, the nerve of the tooth, together with its accompanying artery and vein, are laid bare, and not unfrequently wounded. When this accident arises from the unskilfulness of the operator, the patient is indeed to be pitied; for in such hands the preservation of his teeth is hardly possible, inasmuch as the requisite operation requires great skill and judgment. But the nerve of the tooth is also often laid bare, not only without any fault of the operator, but frequently as an unavoidable consequence of the form and condition of the disease. It may happen when the cavity of the tooth is formed very irregularly, by which the operator is deceived; or it may take place when the caries and inflammation

* This paper we copy from the American Medical Recorder. We understand that the author has settled in this town, and we feel pleasure in the opportunity thus afforded of introducing him to the favourable notice of our readers.

have already penetrated through the whole side of the tooth; in which case, it is better to expose the nerve than to suffer it to remain covered with a portion of dead bony substance, by which it will be continually irritated and ultimately destroyed. It may also occur when the tooth has been previously filled up with metal in an unskilful manner, so that the disease is thereby accelerated in its destructive progress, and a repetition of the operation rendered necessary. In such a case, it is necessary to cut away so much of the substance of the bone as will remove all the carious parts, and to acquire a sufficient hold to retain the metal with firmness in the cavity, in consequence of which the exposure of the nerve becomes unavoidable. Again, the nerve may be exposed when the disease of the tooth is so situated as to require the removal of a very large portion of the tooth, in order that the diseased part may be fairly brought to view, and the cavity cleared of its dead and inflamed parts.

The remedies with which surgery has heretofore supplied us in such cases, appeared to me, from the commencement of my practice, cruel and contrary to reason. All the authors I have yet been able to consult on this subject concur in recommending *the destruction of the nerve of the tooth and its investing membrane.* They advise, for this purpose, the knife, concentrated acids, but particularly the actual cautery.

The pain which is caused by this operation of destroying the nerve, is so intense and protracted, and the idea of it so distressing, that few patients are willing to submit to it. The bare consideration, however, of its producing very intense pain, and appearing very appalling to the patient, would not in itself be a sufficient ground for rejection, if any hope could be entertained of ultimate success. It ought to be the assiduous study, as it is certainly the most important duty of the operator, to overcome these obstacles, by calming the fears of the patient and inspiring that confidence and submission which are alone effected by gentle persuasion, tenderness, and skill. But there are other considerations which render the adoption of this mode of treatment not a little objectionable. The difficulties which occur in the operation itself are by no means to be compared, as an objection to it, to the evil consequences which always come on sooner or later after the operation, and which require particular attention.

The violent irritation which is created by this unnatural operation in the whole nervous system, but more especially in the adjoining nerves and parts, occasions, not unfrequently, in irritable or inflammatory constitutions, in a few days after the operation, an inflammation of the whole mouth, which becomes soon concentrated upon the parts near the affected tooth, where tumefaction and suppuration take place. The pus being discharged from the swelled gums, the patient obtains some relief, but a perfect cure is not accomplished. This can now be effected only by the extraction of the tooth; an operation to which the patient soon flies for relief. In strong and firm constitutions, when this operation of destroying the nerve is performed with neatness and delicacy, these evil consequences sometimes do not show themselves at an early period, nor with much violence. The tumor, after the matter is evacuated, disappears nearly altogether, and leaves nothing but a little hardness. Through this indurated spot the matter issues, which usually collects at the point of the root, and works its passage outwards through the thinnest side of the socket. The irritation of the dead tooth now keeps up a constant, though scanty, discharge through the opening in the indurated spot just mentioned. A portion of the matter collected at the root is absorbed by the lymphatics, and, by irritating the glands in its passage through them, it destroys the healthy secretions of the mouth, which also act as a morbid irritant, both upon the other teeth and upon the stomach, and through this organ, upon the whole system. I have known a single tooth, which has been treated in this manner, become the cause of general disorder to the system, and of all the teeth in the mouth.

A tooth which has been deprived of its vitality by the destruction of its nerve, acts upon the parts with which it is in immediate contact as a foreign dead body. It produces all the evil effects which are usually the consequences of a dead root of a tooth, but in an infinitely greater degree. From the moment a tooth is deprived of life, it becomes a useless and intrusive part in the animal economy, and causes an irritation with which the whole constitution sympathises. In the beginning, the suppuration at the root of a tooth exists in the fasciculus of the nerve, and extends afterwards to the cord of the nerve. The progress of the disease opens a way for the discharge of the matter through the canal of the root. If, therefore, a tooth, which has been treated after the above plan, be filled up with metal, the natural opening for the discharge of the matter is thereby obstructed, and the pus being thus confined and accumulated, works its way through the side of the socket, and produces a fistulous opening, which can only be remedied by extracting the tooth.

Whilst I beg leave to refer the reader to Case I. at the end of this article, for an illustration of the preceding practice, I trust enough has been said to convince him of the impropriety of destroying the nerve of the tooth by such an operation, and that it is a practice which ought to be exploded.

I hope I may now be permitted to detail my own method of operating in cases where the nerve of the tooth has become exposed, which I have practised during eight years past with satisfactory results.

In treating a case of the kind under consideration, I have always held it a principal object to preserve the life of the lining membrane, and thus to save the life of the whole tooth.

My indications for the attainment of this purpose are—

1. To put a stop to the caries, and consequently to prevent the irritation upon the internal membrane of the tooth.

2. To suppress the hæmorrhage, and cure the wound of the membrane, if it be wounded.

3. To protect the membrane artificially against the action of all foreign or external agents.

To obtain the first of these objects, I cut away all the unsound or dead parts of the tooth, so that every part of the carious cavity be sound, firm, and white. I give the cavity the best possible form for the reception of the metal and its firm retention. I next wash it out with a little lock of cotton, fastened to a straight elastic probe, dipped in warm water. The cavity must be very carefully freed from the small pieces of bone that may stick to it.

If the lining membrane is not wounded, I immediately plug the cavity with metal; but, if there exist a wound and hæmorrhage, I resort to the treatment for the second indication, by which I endeavour to put an immediate stop to the bleeding, and to cure the wound. For this purpose, I was for some time, in the commencement of my practice, in the habit of employing mild acids and styptics; but I did not find those applications answer any good purpose. The first act destructively on the surrounding parts, and the second were not sufficiently certain in their operation. I therefore soon abandoned such remedies, and resorted to the actual cautery. By this application I readily effect an artificial cicatrization of the wound and a stoppage of the hemorrhage.

I require for the operation the following apparatus:—1. A thin iron wire, fastened to an ivory handle. The extremity of this wire I file to about the thickness of the exposed surface of the nerve; and bend the wire in such a manner as to enable me to touch the exposed part of the membrane, without coming in contact with any other part of the mouth. 2. A tallow candle, with a thick wick. I direct the patient to discharge all the saliva he may have in his mouth; and then let him incline his head backwards against the head-support of my operation chair. I put the candle into his left hand, and direct him to hold it so that the flame of it may be in a position horizontal with his mouth, and about eight inches from it; I now place myself on the right side of the patient, and, holding his lips with my left hand, so that the instrument may not touch them, I again dry the cavity as perfectly as possible with a lock of cotton fastened to the point of the cauterising wire. Having effected this, I throw away the cotton from the extremity of the wire, and make it red-hot in the flame of the candle. With the wire thus heated I touch the denuded part very rapidly, so that its surface forms an eschar; without, however, suffering it to penetrate deeply into the substance of the bone or the cavity, for this would inevitably bring on suppuration and destruction of the nerve. The nerve must be touched very quickly, and the cautery be perfectly *red* hot. It is sometimes necessary to apply it two or three times before the parts are sufficiently cauterised. When the cautery is red hot it acts suddenly, and almost entirely without pain: but, when it is merely hot, much pain and inflammation is generally produced.

This operation is indeed so little painful, that I have been solicited by my patients to repeat it; although before, they required no little persuasion to induce them to suffer its application. When the hæmorrhage is arrested in this way, and an artificial cicatrix formed, I then leave the further healing altogether to nature, and only caution my patient against such things as might interfere with her salutary operations.

Air is among the most injurious external agents, when conjoined with moisture, upon an exposed nerve. If the nerve of the tooth is long exposed to the influence of these agents, its inflammation, and consequent destruction, is almost inevitable. I terminate, therefore, the operation, by passing to the third indication; which is to protect the nerve against external injurious impressions, by filling up the cavity of the tooth with metal. For this purpose I wash the cavity, as before the cauterisation, with warm water; I carefully remove every particle of the ashes or matter that may have been left by the cauterisation, taking great care not to wound the membrane again.

The nerve, which before cauterisation, assumes a fleshy appearance, looks after this operation like a black point. I take care not to disturb this point; for, if the black scar be removed, a new wound will be formed, and bleeding induced. I now take a small plate of very thin lead leaf, and lay it upon the denuded nerve and the immediately surrounding bony parts. I next fill up the whole cavity very carefully with gold. In order that success may attend this operation, it is absolutely necessary to make the proper curative applications with the utmost degree of exactness and care, since the smallest error in this will inevitably bring on a destruction of the life of the tooth, and consequently its loss. Thus, for instance, the whole operation will prove abortive if the smallest particle of dead matter, or inflamed bony substance, is suffered to remain in the cavity. Such foreign dead matters, left in contact with the living tooth, soon acquire corrosive qualities, and act destructively upon the contiguous parts, by irritating and inflaming them. If any particles be allowed to remain in contact with the nerve, it is impossible that the operation can succeed properly. Even the smallest quantity of blood left in the cavity soon becomes corrosive, and prevents the success of the operation. All kinds of moisture must be removed before introducing the metals, as the two contiguous metals might produce galvanic effects if there be any intervening moisture, and thus create a source of irritation and inflammation to the nerve.

When, therefore, the cavity is once completely cleared of the loose particles of matter, and made perfectly dry, the metal should be quickly introduced, without giving it time to become moist again from the natural exhalations in the mouth. The gold should, of course, be pressed as firmly and compactly into the cavity as possible, in order to prevent the insinuation of any moisture under it. It is here that the skill of the operator becomes of the highest importance; for, if he has been successful in preserving the life of the nerve, the duration of the tooth depends on the skilful manner in which it is plugged; and this is one of those operations by which one dentist may have an opportunity of displaying his superiority over another.

In all surgical operations, the ultimate success depends much upon the sanative powers of nature. After the operator has performed his duty with skill, he can only watch the efforts of nature, and assist her in her sanative operations by a due regulation of such circumstances as are calculated to influence her powers.

It may be asked why I cover the nerve with lead? I do so, because I believe that this metal has a cooling and anti-inflammatory effect upon the irritated nerve of the tooth; at least, I conceive it possesses these qualities in a greater degree than gold. When, in the commencement of my practice, I employed gold exclusively, I was but seldom successful in my labours; for inflammation, pain, &c. always soon came on, and obliged me in a short time to remove the tooth entirely. Having been almost uniformly unsuccessful whilst employing gold alone in this operation, I resorted to the use of tinfoil as an experiment, and with this metal my success was evidently greater, though not what I desired it to be; for, even when the operation succeeded with this metal, which was not often the case, it did not remain long a protection to the nerve, because, on account of its thickness, I could use this metal only. In all cases where the tinfoil is used, the tooth is only preserved for a few years; for the saliva dissolves the metal, and, uniting with it, acts as destructively as the cause itself.

On recollecting the cases so commonly reported of leaden bullets, even when rough and battered, having remained for years imbedded in the flesh of soldiers, I was naturally induced to resort to it in this operation. It does not occur to me that a case has been reported of any other species of metal remaining in the body for a long period, without exciting inflammation and suppuration around it. My experience has ever since strengthened the opinion I drew from these facts; and I am now more confident than ever that this substance is less irritating to living parts than any other metal.

I have used the lead under the gold for above eight years, and I feel warranted in saying that seven-eighths of the teeth on which I have operated will be preserved alive.

In almost every case where a course of various operations is necessary for a perfect restoration of the teeth, we find one or more diseased teeth which require this treatment; and these are commonly amongst the most important, as the incisors, cuspidate, bicuspidate, but very seldom the molares. They generally occur in the upper, and rarely in the under jaw. In the conclusion of this article, I take the liberty of drawing the attention of my reader to Case II. for a general illustration of my practice.

After the operation for the disease in question, we must constantly attend to the following circumstances: 1. The prevention of inflammation of the nerve of the tooth. 2. If inflammation has supervened, to endeavour to prevent its terminating in suppuration.

To answer the first object, the patient must guard himself from imprudent exposure to a damp and cold air, or to any sudden transitions from heat to cold, or *vice versa;* he must also be directed to keep his teeth clean by the use of some suitable dentrifice, good brushes, and warm water. If the general diathesis of the patient's constitution be inflammatory, I direct him to bathe his gums with a mixture of equal parts of strong vinegar and warm water every three or four hours. When the tooth becomes painful, I scarify the gums, and promote the bleeding by warm fomentations. After the bleeding has ended, I request him to wash with the above mixture, with the addition of a little honey. If the gums are a good deal inflamed, I apply from two to six leeches, and order an antiphlogistic regimen. When there are signs of disorder or impurities in the primæ viæ, gentle emetics or laxatives must be resorted to. The operator must not suffer himself to be misled by the complaints of the patient, and remove the tooth at once, when he, through pain, desires it. In many instances the pain ends with the operation, but in other cases the tooth becomes occasionally painful during several months. As long, however, as the tooth possesses vitality, there are hopes of a perfect cure. If the tooth, after about three months, has a natural and lively colour, is free from pain, without being insensible or loose, then the cure may be pronounced as complete. The tooth is now secured as long as the metal remains firm in its cavity, and protects the nerve against the action of external agents. If the tooth be loose, of a pale and unnatural colour; the gums red, swelled, suppurating, and painful; ulcers and fistulous opening in the neighbourhood of the tooth; then we may conclude that the nerve is destroyed, and the operation abortive. In this case it is best to extract the tooth.

CASE I.

On the 22d of May, 1818, I was consulted by Mr. C. of Philadelphia, on the subject of his teeth. He informed me that, about fourteen months previously, he had put himself under the care of a dentist, who, on cutting away the carious parts of the left cuspidate, exposed the nerve of the tooth, which he immediately destroyed by the application of the actual cautery. The pain occasioned by this was so excessive, that the dentist could not, conformably to his original intention, proceed to filling up the cavity of the tooth with gold, but was obliged to defer this part of the operation, and to request the patient to return to him again in five days. A violent inflammation and swelling of the gums supervened, which prevented the completion of the operation at the time the patient was requested to return. He now was directed to use emollient washes for his mouth, &c. with a view of reducing the inflammation.

The swelling immediately around the tooth became large and extremely sensible: it soon, however, broke, and discharged a considerable quantity of pus. The pain was now much mitigated, and the patient expected soon to be able to bear the operation of plugging his tooth.

But the swelling did not wholly subside; the tooth was loose, and extremely tender to the touch. Four weeks after the nerve was destroyed, it was still thought improper to proceed with the operation; and the patient was comforted by his dentist with the hope that the inflammation and swelling would not continue long, and that he would soon be in a proper state to have the operation finished.

In this hope, however, he was disappointed. The tooth remained extremely painful, the swelling of the gums increased, which again terminated in suppuration. After having thus suffered for about five months, he became dissatisfied with his dentist, lost all confidence in the resources of art, and determined to rely for relief on the sanative efforts of nature.

Continued pain and distress soon obliged him to abandon this resolution, and again to apply to our art for aid. The eye-tooth, the nerve of which was destroyed by the actual cautery, was loose, and so painful that the slightest pressure of the finger caused the patient to cry out. Its socket was enlarged, and the inflammation had extended to all the alveoli of the upper jaw, from the protracted irritation of the dead tooth. The whole alveolar process was enlarged; and this enlargement was greater in proportion to its proximity to the dead tooth. These parts also were altered from their natural direction, so that the teeth adjoining the dead one separated from it on either side, and, from being considerably pushed forwards, distorted his mouth in a very disagreeable way. The teeth that had been filed were again affected with caries, which indeed was now making more rapid progress than it had done before they were filed. There was much tartar on all the teeth, and more especially on those which were dead, and the adjoining ones. The gums were greatly inflamed, much swelled and spongy, and, upon the slightest pressure of the finger, poured out black blood, mixed with fetid pus. The breath was very offensive; the patient was pale and emaciated, although naturally of a very healthy and florid complexion.

The patient readily submitted to the plan of treatment I proposed to him, which consisted—

1. In removing the inflammation of the bony structure of the jaw. This I effected, in part, by the removal of the general and topical irritating causes. The causes that kept up the local irritation were, more particularly, the dead tooth and the two diseased molares; which therefore I extracted.

2. The removal of the inflammation and suppuration of the periosteum and gums. To this end I removed the tartar carefully from the teeth, and directed the use of a mildly stimulating wash for the mouth, and the subsequent careful cleansing of the teeth.

In three months after this, I found my patient so much better as to enable me to treat the individual disorders of his teeth with perfect safety; and, by a few operations on these, he had the satisfaction of soon recovering, not only the health of his teeth and mouth, but also that of his system generally.

CASE II.

Mr. B——, a very respectable gentlemen of Philadelphia, consulted me about his teeth, on the 12th of November, 1817. They had been operated upon some years before, rather injudiciously; many of them had been filed, cut out, and plugged, and almost all of them were still continuing to decay, and threatened with speedy destruction. All were a little covered with tartar, from which I immediately relieved them by the necessary operation.

Dec. 11th.—Five teeth were again filed, and one of these cut out and plugged with gold. In the latter, the caries having penetrated into the cavity, the nerve was unavoidably exposed: it was, however, not wounded, and I treated it as has been stated above.

March 26th, 1818.—Four teeth were filed, and four were cut out and plugged with gold. In all of them the nerve was exposed, and in one the wounding of it was unavoidable. In this latter case the nerve was cauterised, and treated as directed above.

July 6th.—Two teeth more were filed, and two more were plugged with gold. Both of the latter presented difficulties similar to those lately mentioned, and in both, the nerves being exposed and wounded, the same treatment was required as in March 26th.

August 29th.—One of the teeth thus preserved became rather painful, and formed a small tumor over the fang; and as this tooth, from its situation, was of no great utility, I considered it best to make no attempt for its preservation, and of course removed it, gaining thereby a greater prospect of success for the others. Thus, it will be seen, of seven teeth in the same mouth with denuded nerves, treated according to the method mentioned above, six are now preserved alive, healthy, and useful; and these are, one upper central incisor, one cuspidatus, two bicuspides, one under bicuspis, and one molar.

